# Developing empirically supported theories of change for housing investment and health

**DOI:** 10.1016/j.socscimed.2014.11.043

**Published:** 2015-01

**Authors:** Hilary Thomson, Sian Thomas

**Affiliations:** MRC/CSO Social & Public Health Sciences Unit, University of Glasgow, 200 Renfield St, Glasgow, UK

**Keywords:** Housing, Evidence synthesis, Public health, Systematic review, Socio-economic determinants of health, Healthy public policy, Warmth, Regeneration

## Abstract

The assumption that improving housing conditions can lead to improved health may seem a self-evident hypothesis. Yet evidence from intervention studies suggests small or unclear health improvements, indicating that further thought is required to refine this hypothesis. Articulation of a theory can help avoid a black box approach to research and practice and has been advocated as especially valuable for those evaluating complex social interventions like housing. This paper presents a preliminary theory of housing improvement and health based on a systematic review conducted by the authors. Following extraction of health outcomes, data on all socio-economic impacts were extracted by two independent reviewers from both qualitative and quantitative studies. Health and socio-economic outcome data from the better quality studies (*n* = 23/34) were mapped onto a one page logic models by two independent reviewers and a final model reflecting reviewer agreement was prepared. Where there was supporting evidence of links between outcomes these were indicated in the model. Two models of specific improvements (warmth & energy efficiency; and housing led renewal), and a final overall model were prepared. The models provide a visual map of the best available evidence on the health and socio-economic impacts of housing improvement. The use of a logic model design helps to elucidate the possible pathways between housing improvement and health and as such might be described as an empirically based theory. Changes in housing factors were linked to changes in socio-economic determinants of health. This points to the potential for longer term health impacts which could not be detected within the lifespan of the evaluations. The developed theories are limited by the available data and need to be tested and refined. However, in addition to providing one page summaries for evidence users, the theory may usefully inform future research on housing and health.

## Highlights

• Theories of health impacts of complex interventions are valuable but poorly developed.
• Including socio-economic data may explain why health impacts are observed or not.
• A theory of healthy housing improvement was prepared using systematic review data.
• Quantitative and qualitative data on health and socio-economic impacts were used.
• Warmth, affordability & suitable space provision may mediate health impacts of housing.

## 1 Introduction

Persistent links between poor housing and poor health have been reported in a wealth of cross-sectional studies and point to the potential for housing improvement to lead to health improvement. However, the complex associations between poverty, poor housing, and poor health, make it difficult to ensure adequate control for confounders (Wilkinson, 1999), (Hunt, 1993). This means that, which or how housing conditions cause poor health remains poorly understood. And, perhaps more importantly, it cannot be assumed that investment to improve housing conditions will lead to improved health. Hypotheses around the possible health impacts of housing improvement need to be empirically tested through evaluations which assess changes in health outcomes following housing improvements.

### 1.1 Empirical evidence on health impacts of housing improvement

At the start of 2013 we published an updated systematic review of evaluation studies which assessed the health impacts of housing improvement (Thomson et al., 2013a, 2013b). This review had broad inclusion criteria, and included studies from any time period, any country, any language, and any design, including quantitative and qualitative studies. Studies which had assessed changes in any measure of illness, health, or wellbeing following a housing improvement intervention were included. The full inclusion and exclusion criteria and the methods are reported in the review published by the Cochrane and Campbell Collaborations (Thomson et al., 2013a, 2013b).

Following comprehensive searches of 39 bibliographic databases, covering journal publications and grey literature, along with expert consultation, 39 studies reporting quantitative and/or qualitative data were identified and included in the review. Both the quantitative and the qualitative studies were assessed for levels of bias and internal validity using established methods which were tailored to allow application to this topic (Dixon-Woods et al., 2004), (Effective Public Health Practice Project, 2010). Those studies assessed to have a high risk of bias, or where poor reporting prevented assessment of validity, were not included (*n* = 15) in the final synthesis. The included studies were grouped according to broad intervention type and also important contextual differences. The four intervention categories were (*n* = included studies at low or moderate risk of bias. NB: numbers do not add up to 39 as some studies reported both qualitative and quantitative data):

• Warmth & energy efficiency: post 1985 (Quantitative *n* = 11; Qualitative *n* = 5)
• Rehousing/neighbourhood renewal: post 1995 (Quantitative *n* = 6; Qualitative *n* = 4)
• Provision of basic housing in Low & Middle Income Countries (LMIC): post 1990 (Quantitative *n* = 1)
• Rehousing from slums: pre 1970 (Quantitative *n* = 1)

Few studies reported standardised effect sizes, and the data were not amenable to meta-analysis. The data were, therefore, synthesised narratively and the resulting synthesis focussed on the nature and direction of health impacts rather than calculating an estimated effect size for each outcome. Reports of health impacts were mixed, supporting the earlier assertion that investment to improve housing should not be assumed to lead to health improvements. Although there was no indication of harms to health, few studies reported statistically significant improvements in health following housing improvement. Studies of warmth and energy efficiency interventions suggested that improvements in general health, respiratory health, and mental health are possible. Studies which targeted those with inadequate warmth and existing chronic respiratory disease were most likely to report health improvement (Howden-Chapman et al., 2007; Howden-Chapman et al., 2008). The health impacts following area based programmes of housing-led neighbourhood renewal were less clear. Only one better quality study (low or moderate risk of bias) was identified for each of the LMIC, and the “rehousing from slum” categories, limiting the ability to draw lessons about the health impacts for these categories of interventions. The review concluded that housing improvement which improves thermal comfort in the home can lead to health improvements, especially where the improvements target people living in housing with inadequate warmth and who have chronic respiratory disease. The health impacts of programmes which deliver improvements across areas and do not target according to levels of individual need are less clear. However, where impacts are reported for whole areas the range of impacts may be concealed by the area level mean, and it is possible that some individuals may have experienced significant health impacts.

### 1.2 Underpinning theory for housing improvement and health

Interest in the health impacts of housing improvement assumes that public investment to improve housing conditions has the potential to be part of healthy public policy, i.e. the use of public investment outside health services, to contribute to improved health by addressing one or more socio-economic determinant of health (Milio, 2001) The notion of housing as part of healthy public policy is often implicit within both policy and evaluations of housing improvements. Policy statements which accompany announcements of housing investment have indicated expectations of health improvement as a result of the investment, but do not specify what type of health impacts might be expected, the timescale for impacts, or how the investment might lead to improved health. For example, in a government document for the UK's programme of neighbourhood renewal, New Deal for Communities, the programme vision was stated to be ‘to have lower worklessness: less crime; better health; better skills and better housing’ (Social Exclusion Unit, 2001). Similarly, in evaluations of housing improvement there is often an implicit, rather than explicit, assumption that the housing improvement will lead to exposure to improved housing conditions for residents, and that this exposure has the potential to lead to health improvement within the, often short, timescale of the evaluation. We conducted a brief search for published work theorising the potential for improved housing conditions to lead to improved health but found little. Dunn et al. examined the underlying programme theory for a specific policy providing housing for homeless people with severe mental illness, but this did not incorporate consideration of health impacts (Dunn et al., 2013). While Shaw proposed a preliminary theory which took into account the importance of material factors and the “meaning” of housing for health either directly or indirectly, the model does not focus on mechanisms for housing improvement (Shaw, 2004). Most recently, Gilbertson et al., and also Liddell & Guiney have posited the importance of stress as a pathway through which fuel poverty may impact on health (Gilbertson et al., 2012; Liddell and Guiney, 2014).

The near absence of a theory articulating possible mechanisms through which housing improvement might lead to improved health indicates that assumptions about the potential impacts, both positive and negative, of well intentioned interventions have yet to be made explicit. A black box approach to evaluation which only investigates changes in endpoint outcomes is limited with respect to identifying important explanations for a lack of expected impacts, or unintended harmful impacts. Use of theory in developing and evaluating interventions promotes development of explicit hypotheses about the nature of, and routes to impacts following an intervention, as well as identifying potential mediating factors for the intended impacts of the intervention. Using these theories as frameworks to shape and improve subsequent evaluations is valuable and facilitates further testing and refinement of the theory and the intervention, with the aim of maximising the benefits and mitigating identified harmful effects of the intervention (Chen and Rossi, 1983). Social programme theory has been recommended as a tool to help develop interventions and accompanying evaluations which might generate evidence for healthy public policy, like housing improvement for health (Rychetnik et al., 2002), (Craig et al., 2008).

The systematic review outlined above, did not identify consistent or strong support for the hypothesis that housing improvement leads to health improvement. This may be counterintuitive, and challenge the assumption, that a well intentioned investment to improve living conditions will lead to improved health. The lack of a clear and consistent effect across studies suggests that there are important mediating factors which may interrupt the pathway between intervention and impact. Consideration of one key outcome, in this case health, as a measure of “what works?” is inherently limited, perhaps particularly for complex social interventions, such as housing improvement. It may also be unrealistic to expect health outcomes to improve in the relatively short timescale, often a year or less, of an evaluation. Consideration of only one key outcome ignores the important influences of and interactions between context and outcome (Chen and Rossi, 1983) (Pawson and Tilley, 1997). Such an approach is limited in addressing important questions of how, and why housing improvement will or will not lead to health improvements, and does not contribute to development and refinement of underpinning theories for social interventions like housing improvement. Evaluations of endpoint outcomes, such as health, would have greater explanatory value if they incorporated assessment of changes in proximal outcomes to explore the mechanisms and causal pathway for health impacts.

### 1.3 Socio-economic impacts as pathways to health impacts

Unlike health service interventions, the primary aim of housing improvement is not to improve health. Rather, the rationale or theory underpinning the notion of healthy public policy, including healthy housing investment, is that by improving socio-economic determinants of health, such as living conditions, health will subsequently improve. To help develop and test this theory it is necessary to systematically examine the body of empirical evidence available on whether and how housing improvement impacts on living conditions, and on other socio-economic determinants of health. Using the above systematic review, this paper reports the methods and findings of further systematic interrogation of the housing improvement studies for empirical evidence of impacts on socio-economic determinants of health. The extracted data are presented in a logic model with the aim of developing empirically supported theory of the mechanisms through which housing improvement may lead to health improvement.

## 2 Methods

We conducted a systematic review of the health impacts of housing improvements which was published in early 2013. The methods and findings of this review are outlined above, and full details of the methods are available from the Cochrane Collaboration and Campbell Collaboration online libraries (Thomson et al., 2013a, 2013b). Data on changes in health and socio-economic outcomes were extracted from each study, including quantitative and qualitative data. Data were extracted by one reviewer and checked by a second reviewer, with disagreements resolved by discussion. Socio-economic determinants of health included any data on educational, employment, nutritional, financial, inter-personal relationship, and wellbeing outcomes. Data on changes in housing condition, view of the house, and use of the house were also extracted to allow for examination of mechanisms for subsequent impacts. Details of the study interventions, population and context were also extracted and examined in detail when conducting the synthesis of health impacts. These data were used to identify characteristics which were associated with variations in reported impacts. These data are available in the full reports and were used to shape the conclusions of the synthesis (Thomson et al., 2013a, 2013b).

Following finalisation of the data extraction process, both the quantitative and qualitative data from the better quality studies were mapped onto single page logic models for two of the intervention categories: “Warmth & energy efficiency: post 1985”; and “Rehousing/neighbourhood renewal: post 1995”. Due to issues of relevance, and quantity of studies at low/moderate risk of bias, logic models were not prepared for the housing intervention categories “Provision of basic housing in Low & Middle Income Countries (LMIC): post 1990” and “Rehousing from slums: pre 1970”. Where links between impacts and outcomes were reported in the data, these were mapped using a solid line to indicate that the link was supported by data. Where no link between two outcomes was specifically reported but was implied, a dotted line was used to indicate an implicit or assumed link. For example, improved housing conditions were assumed to be a forerunner of the improvements in respiratory outcomes reported, but no actual link was reported so a dotted line was used. The logic model lines use arrows moving from left to right to indicate the temporal direction from one impact as a forerunner to another impact. An indication of the data source, study name, was included in the logic model to ensure transparency of the resultant evidence map. The logic models were prepared independently by two reviewers, and a final version of the logic model for both housing intervention categories was prepared to reflect areas of agreement between the two reviewers.

Using the data from the two logic models a further logic model reflecting the areas of commonality across both intervention categories (“Warmth & energy efficiency: post 1985”; and “Rehousing/neighbourhood renewal: post 1995”) was also prepared to provide a broader level model of pathways between housing, housing improvement and health.

## 3 Results

**Fig. 1:**
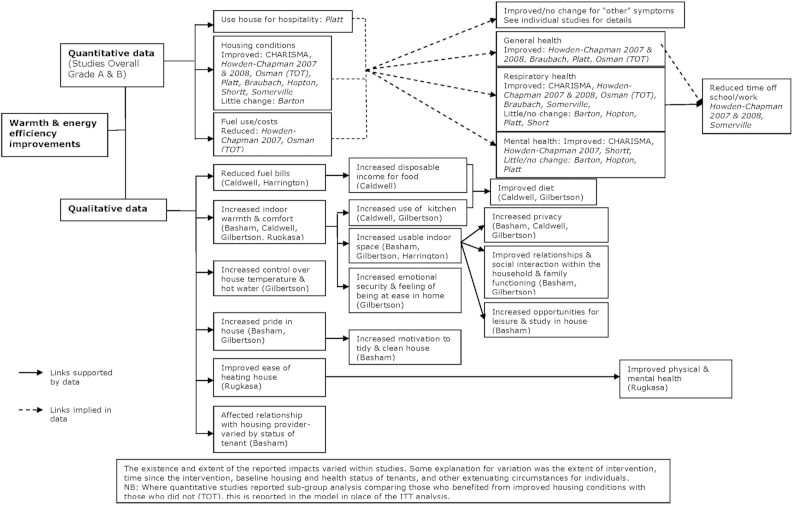
Logic model mapping reported quantitative & qualitative impacts following warmth & energy efficiency improvements to housing.

**Fig. 2:**
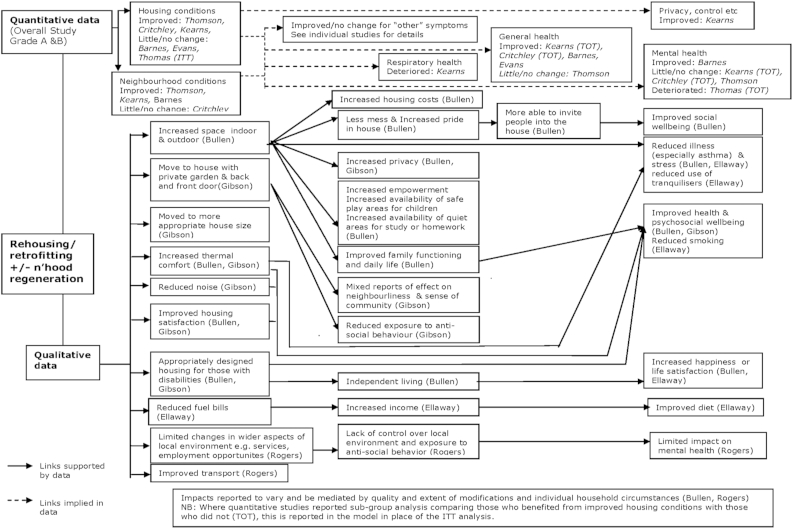
Logic model mapping reported quantitative & qualitative impacts following housing-led neighbourhood renewal.

Data from the 23 better quality (assessed to be at low or moderate risk of bias) studies were used to develop the logic models. This included data from 17 quantitative papers, and nine qualitative papers or reports. The studies reported a diverse range of health and socio-economic outcomes. Although some outcomes were similar, it was rare for the same outcome to be reported in more than one study. Outcomes were grouped into broad domains, for example respiratory outcomes, dietary outcomes, relationships, etc. The resulting logic models for warmth & energy efficiency housing improvements, and housing-led neighbourhood renewal are presented in Figs. 1 and 2.

### 3.1 Warmth & energy efficiency housing improvements: available data

Data from 14 studies were included in the evidence synthesis of the impacts of warmth and energy efficiency measures. Two of the quantitative studies were conducted in New Zealand, (Howden-Chapman et al., 2007; Howden-Chapman et al., 2008) and the remaining quantitative and qualitative studies were conducted in the UK. Nine studies reported only quantitative data, (Hopton and Hunt, 1996; Somerville et al., 2000; Barton et al., 2007; Howden-Chapman et al., 2007; Platt et al., 2007; Shortt and Rugkasa, 2007; Braubach et al., 2008; Howden-Chapman et al., 2008; Lloyd et al., 2008; Osman et al., 2010; Woodfine et al., 2011) and three studies reported only qualitative data (Caldwell et al., 2001; Basham et al., 2004; Harrington et al., 2005; Gilbertson et al., 2006; Shortt and Rugkasa, 2007). Two studies reported both quantitative and qualitative data: Rugkasa et al. (quantitative) (Rugkasa et al., 2004) was linked with Shortt et al. (qualitative) (Shortt and Rugkasa, 2007), and Barton et al. (quantitative) (Barton et al., 2007) was linked with Basham et al. (qualitative) (Basham et al., 2004).

### 3.2 Warmth & energy efficiency housing improvements: impacts on housing conditions and housing related issues

Improvements in physical housing conditions, including improvements in warmth, were reported in both the quantitative and qualitative studies. The extent and objective measurement of changes in housing conditions were rarely reported, instead these reports relied on occupants' own assessment of the change. There were reports of improved warmth in all but one of the studies. In one study, the occupants reported little change.

Data from the qualitative studies allowed for incorporation of a wider and more specific range of changes in housing related factors than was possible using only the quantitative data. The range of housing related impacts reported by residents to be linked to the housing improvement varied across the studies, they included: reduced fuel bills, increased pride in the house, greater control over domestic temperature, and an improved relationship with the landlord. In three studies, greater warmth was linked to an increase in usable indoor space in the home (Basham et al., 2004; Harrington et al., 2005; Gilbertson et al., 2006).

### 3.3 Warmth & energy efficiency housing improvements: links to socio-economic determinants of health

Qualitative reports of increased usable space were reported to lead to greater levels of privacy within the home, (Caldwell et al., 2001; Basham et al., 2004; Gilbertson et al., 2006) as well as improved relationships and interaction between household members (Basham et al., 2004; Gilbertson et al., 2006). There were also reports of greater opportunities for studying at home, and socialising and offering hospitality in the home, (Basham et al., 2004; Platt et al., 2007) as well as greater use of the kitchen which was linked to improvements in diet in two studies (Caldwell et al., 2001; Gilbertson et al., 2006). In one study, increased disposable income, as a result of lower fuel bills, was reported to facilitate improvements in diet (Caldwell et al., 2001).

There were few quantitative data on socio-economic impacts. Three studies presented quantitative data reporting a reduction in time off work or school following the warmth improvements (Somerville et al., 2000; Howden-Chapman et al., 2007; Howden-Chapman et al., 2008); (Free et al., 2010). In one study the reduction in absences due to asthma was statistically significant, but reductions in absences due to other causes were not statistically significant (Somerville et al., 2000). This suggests that the reduced absences were linked to improvements in respiratory health which were also reported in the qualitative component of these studies.

### 3.4 Warmth & energy efficiency housing improvements: links to health impacts

Each of the quantitative studies assessed changes in at least one health outcome. The synthesis suggests that there were improvements in general health, respiratory health, and mental health. However, the health improvements were often small, and varied across the studies, with some studies reporting little evidence of any health impact. The evidence synthesis concluded that warmth and energy efficiency improvements can lead to health improvements, in particular general and respiratory health. However, improvements are most likely where the warmth improvements are delivered to households with inadequate warmth and where household member(s) suffer from existing chronic respiratory disease, for example asthma. There were few reports in the qualitative data suggesting that residents made direct links between the housing improvement and health impacts. One qualitative study reported that residents linked improvements in health to increased ease in heating their house following the improvement (Rugkasa et al., 2004).

### 3.5 Housing-led neighbourhood renewal: available data

Data from nine studies were included in the evidence synthesis of the impacts of rehousing or housing led neighbourhood renewal. One study was conducted in New Zealand, (Bullen et al., 2008) and the remaining studies were all conducted in the UK. Four studies reported only quantitative data, (Evans and Layzell, 2000; Barnes, 2003; Critchley et al., 2004; Thomson et al., 2007) and three studies reported only qualitative data (Ellaway et al., 2000; Bullen et al., 2008). Two studies reported both quantitative and qualitative data: a study by Kearns et al. (quantitative) (Kearns et al., 2008) was linked with Gibson et al. (qualitative) (Gibson et al., 2011); and Thomas et al. (quantitative) (Thomas et al., 2005) was linked with Rogers et al. (qualitative) (Rogers et al., 2008).

### 3.6 Housing-led neighbourhood renewal: impacts on housing conditions and housing related issues

Improvements in physical housing conditions were reported in three of the quantitative studies (Critchley et al., 2004; Thomson et al., 2007; Kearns et al., 2008). But another three quantitative studies reported little or no change in housing conditions (Evans and Layzell, 2000; Barnes, 2003; Thomas et al., 2005). The type of physical housing improvements reported in the qualitative studies was wide ranging, reflecting the breadth of measures incorporated within the catch-all category of “rehousing and housing-led neighbourhood renewal”. In addition to reports of reduced fuel bills, (Ellaway et al., 2000) improved thermal comfort, and increased housing satisfaction and pride in their house, (Bullen et al., 2008; Gibson et al., 2011) there were reports of living in a house which was a more appropriate size or design (Bullen et al., 2008; Gibson et al., 2011). In some cases this was the result of a house extension to increase space, and in others it was the result of moving to a smaller house. In some cases there had been improvements in housing design for people with disabilities, and for others there had been a change in housing type and design, for example moving from a flat to a house with a back and front door, and a garden (Bullen et al., 2008; Gibson et al., 2011).

Housing-led neighbourhood renewal typically involves other changes to the immediate external housing environment, and investment in local infrastructure and services. This was raised by participants in one qualitative study. Residents reported improved transport, but also used the opportunity to comment on the limited changes effected by the investment (Rogers et al., 2008).

### 3.7 Housing-led neighbourhood renewal: links to socio-economic determinants of health

In one New Zealand study which addressed overcrowding by extending homes, increased space was reported to be linked to increased housing costs (Bullen et al., 2008). Beneficial impacts of increased space were also reported, for example, increased privacy and opportunities to study and offer hospitality in the home, better family functioning, and less mess (Bullen et al., 2008; Kearns et al., 2008; Gibson et al., 2011). Housing design was also reported to have impacts. Residents moving from a flat to a house with a front and back door, and a private garden reported reduced exposure to anti-social behaviour, but there were mixed reported of effects on neighbourliness and a sense of community (Gibson et al., 2011). Housing design measures to improve access and utility for residents with disabilities were reported to improve levels of independence (Bullen et al., 2008).

### 3.8 Housing-led neighbourhood renewal: links to health impacts

Small improvements in measures of general health were reported in some quantitative studies (Evans and Layzell, 2000; Barnes, 2003; Critchley et al., 2004; Kearns et al., 2008) following housing-led neighbourhood renewal, however one study reported no change (Thomson et al., 2007). There was little evidence from the quantitative studies of improved mental health, and one study assessing changes in respiratory health reported a small deterioration (Kearns et al., 2008) In the qualitative data, improvements in health and health behaviours were linked to improved housing conditions. Increased space was reported to lead to reduced illness and stress, (Bullen et al., 2008) and reduced use of tranquilisers, (Ellaway et al., 2000) and provision of appropriately designed housing for those with disabilities was linked directly to improved health and wellbeing (Bullen et al., 2008; Gibson et al., 2011). Improvements in thermal comfort and housing satisfaction, and reduced noise were reported to be linked to improvements in health and wellbeing.

Links between reported changes in broad socio-economic determinants of health and changes in health outcomes were reported by some residents in the qualitative studies. In one study improved family functioning, and being able to invite people in to the house was linked to improvements in wellbeing (Bullen et al., 2008). Another study reported that increased disposable income facilitated improvements in diet (Ellaway et al., 2000).

### 3.9 Overall model of housing improvement & health

**Fig. 3:**
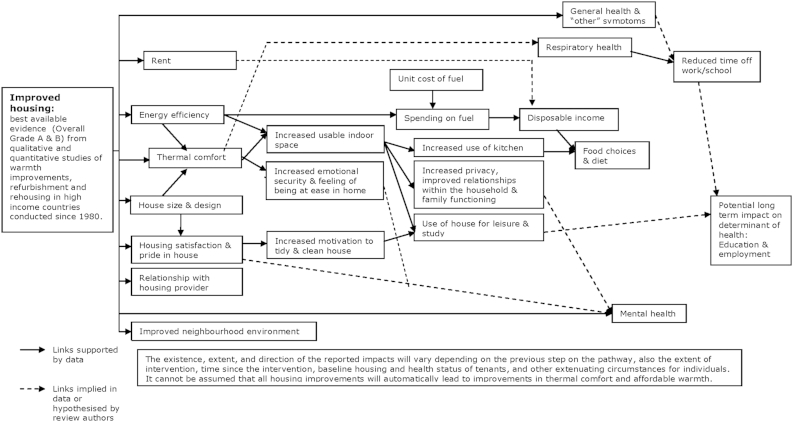
Overall logic model mapping reported health and socio-economic impacts, and potential pathways to health following housing improvement.

The two categories of housing improvement (warmth & energy efficiency, and housing-led neighbourhood renewal) covered by the evidence maps or logic models differed, not only between intervention categories, but also across studies within the same category. Nevertheless, there were areas of commonality across the two categories, notably the inclusion of improvements to warmth and thermal comfort as well as commonality in the impacts on housing factors. The areas of commonality were mapped onto a third logic model (Fig. 3) to present a one page visual of generic impacts and pathways between elements of improved housing conditions and health.

Based on the empirical data identified in the review, the key housing and housing related outcomes affected by housing improvement appear to be: size and usable space; design; thermal comfort; costs (including fuel & rent); housing satisfaction & control over living environment; relationship with housing provider; and neighbourhood environment. Each of these, except changes in the neighbourhood, was linked to changes in possible determinants of health and is listed in Box 1. The main socio-economic determinants of health reported to be affected by changes in these housing outcomes are income, and relationships within the household. There were some reports of changes in diet and eating patterns where residents felt more able to use the kitchen.

Most of the studies were conducted in the UK where improved thermal comfort usually means increased warmth. The model developed (Fig. 3) uses the term thermal comfort recognising that housing should protect residents from extremes of temperature, both hot and cold. Housing costs, namely rent and fuel costs, were sometimes impacted on and these were reported to be important changes. Where fuel bills were reduced this led to an increase in disposable income which was reported to affect food choices and diet. However, the unit cost of fuel, and weather are important determinants of fuel bills. This, together with the potential for improved efficiency to result in reduced costs may lead to “take back” and increased energy consumption, (Alcott, 2005) means that energy efficiency measures cannot be assumed to lead to reductions in fuel bills. Rents often increased following housing improvement, for many low income tenants major increases were often covered by welfare provision, though this cannot be assumed. Those on low incomes who are not eligible for housing benefit may be adversely affected by increases in rent. Increased housing satisfaction was reported to lead to increased care of the house as well as use of the house to offer hospitality.

The direction of effect on the outcomes listed in Box 1 was not always the same, specifically for changes in domestic space, where either increases or decreases could benefit different households. For example, a move to a smaller house was reported to be beneficial for elderly residents. While for those with families, an increase in space was reported to be beneficial. This suggests that increased space is not a universal benefit, rather changes to household space should be tailored to meet household needs. The changes in usable space were facilitated partly by physical changes to space, but also by installation of heating systems which provided affordable warmth. Residents reported being able to heat more of the home and being able to use more of the house following the improvement. This was linked to subsequent improvements in domestic relationships, as well as increased opportunities for privacy, studying, and leisure in the home.

Quantitative data on the health impacts of housing improvement are mixed. Reported improvements in respiratory health appear to be linked to improvements in thermal comfort. While improvements in mental health and wellbeing would appear to be linked to other aspects of housing improvement, such as increased housing satisfaction, increased usable space, and improved domestic relations. However, these links are not confirmed by quantitative assessments of impacts on mental health; this is due to a lack of relevant data rather than an absence of a reported effect.

### 3.10 Influence of variation in implementation and population characteristics

The data used to develop these theories are drawn from a diverse range of studies. However, the heterogeneity can help identify key factors related to the presence or absence of health impacts, or explain variation in the extent of reported health impacts.

Critically, the studies varied greatly with respect to the nature and extent of housing improvement delivered, and also the potential to benefit within the study sample. For example: interventions were sometimes tailored according to individual need; in some studies eligibility to receive the housing improvement was based on housing condition and health status; while in others eligibility only involved residing in the local area. Variation in implementation is another key determinant of the extent of housing improvement experienced by the residents. Interventions may not have been delivered or used as hypothesised. For example, a central heating system may not have been installed properly, or may not have been used by the resident. These issues varied between and within studies, and were not clearly reported. This makes it difficult to make an accurate assessment of the influence of these factors. Nevertheless, the comparison of warmth improvement studies which targeted people living with inadequate warmth and with existing chronic respiratory disease, with studies which did not target by housing need or health status highlights the value of targeting those with the greatest potential to benefit. The synthesis of quantitative health impacts concluded that housing improvement can lead to health improvement, but that the potential to benefit depends on a number of factors. These include, delivery of meaningful improvements in thermal comfort to residents, targeting of those with poor health and inadequate warmth, and no detrimental impacts on disposable income due to housing costs (including fuel costs).

## 4 Discussion

Data from the systematic review were used to prepare a single empirically based theory of how improved housing conditions may lead to longer term health impacts. Although both quantitative and qualitative data were included, there were no quantitative data on intermediate outcomes. In addition to an overall theory of pathways between housing improvement and health, two specific theories for warmth and energy efficiency improvements, and housing-led neighbourhood renewal were prepared. Despite little clear evidence of health impacts, a broad range of short term socio-economic impacts were reported in the qualitative data following housing improvement. These short term impacts may be the first step on a pathway to health impacts which are the result of housing improvement but which may not emerge within the relatively short timeframe of an evaluation.

### 4.1 Empirically based theory of housing improvement & health

The most common impacts of housing improvement on housing related factors included changes in housing conditions such as warmth, housing costs, usable domestic space, use of the home, and attitudes and feelings about the home. The extent of impact on housing factors varied and was largely dependent on issues of implementation, and the potential for tangible improvement in housing conditions. These factors were also susceptible to uncontrollable external mediators such as wider economic changes, for example changes in unit fuel prices. The changes in housing factors were reported to be linked to subsequent changes, most commonly an increase in disposable income following a reduction in housing costs, (Caldwell et al., 2001; Harrington et al., 2005) and improved studying and leisure opportunities, as well as improved domestic relations facilitated by an increase in usable domestic space (Basham et al., 2004; Harrington et al., 2005; Gilbertson et al., 2006; Bullen et al., 2008). A small number of participants reported that these socio-economic impacts were linked to subsequent impacts on health or wellbeing, e.g. improved family functioning leading to improved wellbeing (Ellaway et al., 2000; Bullen et al., 2008).

The data from the review linking socio-economic impacts to health impacts is limited, and is largely based on a small number of participants within the qualitative studies. This may be insufficient to confirm that the socio-economic impacts act as a step on the pathway towards health impacts. However, in combination with evidence of associations from cross-sectional surveys, the data from the review can lend support for hypotheses that socio-economic impacts of housing improvement might lead to health impacts, and facilitate improvements in health promoting behaviour in the longer term. For example, increased disposable income may improve wellbeing through reduced financial stress (Nettleton and Burrows, 1998; Lorant et al., 2003). Positive impacts on feelings of control over one's living environment and housing satisfaction may also be linked to improvements in overall life satisfaction, mental health and wellbeing (Fried, 1984; Phillips et al., 2005). Increased usable space may lead to a number of impacts, which may be associated with subsequent health benefits. Increased privacy may improve domestic relations which may promote improved mental health. Facilitation of studying through increased space and privacy may also lead to improved educational achievement, (Mueller and Tighe, 2007) and impact on earning and employment opportunities, potentially leading to improved health through increased income (Lahelma et al., 2004). There is also quantitative data from the studies included in the review, reporting that improved respiratory health following housing may lead to reduced absences from school (Somerville et al., 2000; Howden-Chapman et al., 2007; Howden-Chapman et al., 2008); (Free et al., 2010). This may lead to improved educational achievement and socio-economic status, and consequently to health benefits in the longer term. Provision of space appropriate to needs may also reduce the health risks of overcrowding (Office of the Deputy Prime Minister, 2004). Although the systematic review did not find consistent evidence to conclude that warmth improvement measures can be assumed to be followed by health improvement, the review concluded that measures which were targeted at those in most need were most likely to lead to improved health. This suggests that delivery of tangible improvements in thermal comfort is likely to lead to improved health, in particular respiratory health, and this improvement may occur within a few months of the intervention (Howden-Chapman et al., 2007; Howden-Chapman et al., 2008). Exposure to extreme temperatures, either cold or heat, is a well established threat to health, especially for vulnerable groups such as the elderly and those will existing cardiac or respiratory conditions (Rudge, 2011). This established association, in combination with evidence from the intervention studies, enhances support for the theory that housing which provides an affordable level of thermal comfort may improve health and prevent adverse health effects of cold, especially among the most vulnerable.

### 4.2 Strengths & weaknesses of the methods & emergent theory

The logic models of the reported impacts provide an accessible visual map of the best available evidence, both quantitative and qualitative, which has been identified and appraised in a rigorous and comprehensive systematic review. The qualitative data were particularly valuable in reporting a range of outcomes which were not pre-specified, and also allowing links between outcomes which were reported by residents to inform the model. The logic models promote transparency of the resulting theory by providing details of the source or study name for each listed impact. Moreover, only the better quality studies have been included in the models, this reflects the data that were prioritised in the synthesis and used to shape the conclusions of the systematic review. There is, of course, considerable detail for each included study and reported impact which is not presented in the logic models. These data were included in a lengthy narrative synthesis published as part of the original systematic review. The logic models not only help develop a theory of housing improvement and health, but also aid evidence users by providing a visual summary of complex data which when narratively synthesised was over 10,000 words long.

Despite mapping all the best available evidence from intervention studies, the resulting theory of housing improvement and health impacts remains limited by the characteristics of the available data. This means there is no overall estimate of size and the timescale of impacts is quite short term (ranging from three months to three years after the intervention). What has been produced represents a rudimentary indication of the nature and direction of possible impacts and pathways to impacts, with the accompanying narrative synthesis providing more detail where required (Thomson et al., 2013a, 2013b). While based on a comprehensive search for empirical studies the theory is limited to a relatively small group of studies and what these studies selected to assess, as well as the inclusion criteria of the review. The review only included changes in housing structure, and did not consider issues of housing tenure, or provision of education or stand alone equipment to reduce domestic hazards, such as lead or fall hazards. Although the qualitative data allow for unforeseen impacts to emerge, the space required in Figs. 1 and 2 to include the wide range of impacts reported in the qualitative data may overemphasise the importance of the qualitative data compared to the larger quantitative studies. Moreover, there may be a lack of perspective within the qualitative data with respect to the wider importance of impacts reported by small numbers of individuals. In addition, the inclusion of an impact reported by one or two people in a small qualitative study may overstate that importance and transferability of the impact. A broad range of housing improvements are incorporated into the evidence map, as well as incorporating a diverse range of populations and contexts. While this heterogeneity can usefully highlight some explanations for variations in reported impacts, for example targeting of housing improvement to those in most need, there is limited scope for detailed comparison across studies. As mentioned earlier, poor reporting of implementation and changes of exposure to poor housing conditions prevents accurate assessments of the key components or the extent of housing improvement needed to effect change.

### 4.3 Contribution of empirically supported theory of housing improvement and health

Despite these limitations, this one page visual map of the best available evidence provides a substantive contribution to a theory of housing improvement and health. Incorporation of all impacts and reported links, along with the principal outcome of interest, namely health, adds considerable value to the original evidence synthesis. While still rudimentary, the resultant model provides empirical support for key factors which can mediate the link between housing improvement and health. This information could help maximise the potential health gain from future housing investment. The emergent theory may also usefully inform future research. The mapped evidence highlights the gaps in current knowledge, and draws attention to the many unanswered questions. The theory needs to be further tested and refined to improve its specificity and reliability. Using this preliminary theory could help inform future evaluations with respect to developing pre-specified hypothesis, and inclusion of primary and secondary outcomes.

## 5 Conclusions

The health impacts of housing improvement may not be assumed. However, best available evidence indicates that housing which is an appropriate size for the householders and is affordable to heat is linked to improved health, and may promote improved social relationships within and beyond the household. In addition, there is some suggestion that provision of adequate, affordable warmth may reduce absences from school or work. The key housing outcomes reported to be affected by housing improvement are living space & design; thermal comfort; housing costs; and attitudes to the home. These have sometimes been linked to impacts on socio-economic determinants of health such as income, education, and employment outcomes. Despite the lack of clear health impacts within the timescale of existing evaluations, reported impacts on socio-economic determinants of health point to the potential for longer term impacts on health following housing improvement.

Socio-economic impacts that accompany housing improvement, may act as mediating influences on health, and this may explain why expected health impacts are, or are not, observed, or why adverse impacts sometimes occur following housing improvement. Systematic mapping of impacts on health and socio-economic outcomes provides a helpful digest of the evidence for health impacts of housing improvement. Incorporation of both quantitative and qualitative data highlights the value of including both data types; the qualitative being particularly valuable in illustrating possible mechanisms for impacts. Moreover, mapping these data provides a preliminary theory of housing improvement and health. Accumulation of further evidence from future evaluations as well as syntheses of other sources of evidence which use this theory to prioritise outcomes are now needed to contribute further iterations and refinements. Adoption of this theory based approach will ultimately improve what is known about, and how to maximise, the potential health impacts of housing and housing improvement.

## Funding

Both authors were funded by the Chief Scientist Office at the Scottish Government Health Directorate as part of the Evaluating Social Interventions programme at the Medical Research Council & Chief Scientist Office Social & Public Health Sciences Unit, University of Glasgow (U.130059812).

## Box 1 Key outcomes reported by residents to be affected by housing improvement.

Domestic space & design appropriate to needs.

Thermal comfort.

Housing costs.

Attitudes to home-satisfaction & control over living environment.
